# Supplementary material for: Metazoan evolution of glutamate receptors reveals unreported phylogenetic groups and divergent lineage-specific events
Source: eLife. 2018 Nov 22;7:e35774. doi: 10.7554/eLife.35774 (PMC6307864; doi:10.7554/eLife.35774)
Supplement: Figure 1—source data 4. [file elife-35774-fig1-data4.pdf]

Figure 1 – figure supplement 6. Summary of MolProbity results.

All-Atom Contacts	Clashscore*, all atoms:	83.89		16th percentile* (N=37, 3Å - 9999Å)
Protein Geometry	Poor rotamers	28	3.73%	Goal: <0.3%
	Favored rotamers	674	89.87%	Goal: >98%
	Ramachandran outliers	13	1.43%	Goal: <0.05%
	Ramachandran favored	837	92.18%	Goal: >98%
	MolProbity score^	3.31		79th percentile* (N=342, 3.25Å - 3.85Å)
	Cβ deviations > 0.25Å	5	0.60%	Goal: 0
	Bad bonds:	2 / 7190	0.03%	Goal: 0%
	Bad angles:	154 / 9785	1.57%	Goal: <0.1%
Peptide Omegas	Cis Prolines:	1 / 35	2.86%	Expected: ≤1 per chain, or ≤5%
	Cis nonProlines:	2 / 874	0.23%	Goal: <0.05%
	Twisted Peptides:	1 / 909	0.11%	Goal: 0
Low-resolution Criteria	CaBLAM outliers	27	2.98%	Goal: <1.0%
	CA Geometry outliers	18	1.98%	Goal: <0.5%

All-Atom Contacts	Clashscore*, all atoms:	65.68		24th percentile* (N=37, 3Å - 9999Å)
Protein Geometry	Poor rotamers	7	0.93%	Goal: <0.3%
	Favored rotamers	737	98.27%	Goal: >98%
	Ramachandran outliers	7	0.77%	Goal: <0.05%
	Ramachandran favored	858	94.49%	Goal: >98%
	MolProbity score^	2.66		96th percentile* (N=342, 3.25Å - 3.85Å)
	Cβ deviations > 0.25Å	0	0.00%	Goal: 0
	Bad bonds:	296 / 7188	4.12%	Goal: 0%
	Bad angles:	217 / 9781	2.22%	Goal: <0.1%
Peptide Omegas	Cis Prolines:	0 / 35	0.00%	Expected: ≤1 per chain, or ≤5%
	Twisted Peptides:	19 / 909	2.09%	Goal: 0
Low-resolution Criteria	CaBLAM outliers	38	4.19%	Goal: <1.0%
	CA Geometry outliers	20	2.21%	Goal: <0.5%

Summary of MolProbity results for the 3D model of full-length GluE1_Bla. The model comprises residues Met1 to Lys910 and was generated by RaptorX using PDB entry 3kg2 as template

(automatically selected), and further refined with ModRefiner. The tables presented indicate model quality before and after refinement, respectively. Note the improvement of the model upon refinement, as indicated by the clashscore, Ramachandran, and overall MolProbity values.

*Clashscore is the number of serious steric overlaps (> 0.4 Å) per 1000 atoms. 100th percentile is the best among structures of comparable resolution; 0th percentile is the worst. For clashscore the comparative set of structures was selected in 2004, for MolProbity score in 2006.

^MolProbity score combines the clashscore, rotamer, and Ramachandran evaluations into a single score, normalized to be on the same scale as X-ray resolution.

All-Atom Contacts	Clashscore*, all atoms:	103.39		10th percentile* (N=37, 3Å - 9999Å)
Protein Geometry	Poor rotamers	34	3.80%	Goal: <0.3%
	Favored rotamers	779	87.04%	Goal: >98%
	Ramachandran outliers	25	2.41%	Goal: <0.05%
	Ramachandran favored	947	91.41%	Goal: >98%
	MolProbity score^	3.43		73rd percentile* (N=342, 3.25Å - 3.85Å)
	Cβ deviations >0.25Å	16	1.66%	Goal: 0
	Bad bonds:	2 / 8392	0.02%	Goal: 0%
	Bad angles:	206 / 11361	1.81%	Goal: <0.1%
Peptide Omegas	Cis Prolines:	1 / 43	2.33%	Expected: ≤1 per chain, or ≤5%
	Cis nonProlines:	1 / 994	0.10%	Goal: <0.05%
	Twisted Peptides:	2 / 1037	0.19%	Goal: 0
Low-resolution Criteria	Dablam outliers	54	5.22%	Goal: <1.0%
	CA Geometry outliers	20	1.93%	Goal: <0.5%

All-Atom Contacts	Clashscore*, all atoms:	54.48		45th percentile* (N=37, 3Å - 9999Å)
Protein Geometry	Poor rotamers	6	0.67%	Goal: <0.3%
	Favored rotamers	872	97.43%	Goal: >98%
	Ramachandran outliers	18	1.74%	Goal: <0.05%
	Ramachandran favored	960	92.66%	Goal: >98%
	MolProbity score^	2.67		96th percentile* (N=342, 3.25Å - 3.85Å)
	Cβ deviations >0.25Å	0	0.00%	Goal: 0
	Bad bonds:	331 / 8391	3.94%	Goal: 0%
	Bad angles:	223 / 11359	1.96%	Goal: <0.1%
Peptide Omegas	Cis Prolines:	0 / 43	0.00%	Expected: ≤1 per chain, or ≤5%
	Twisted Peptides:	17 / 1037	1.64%	Goal: 0
Low-resolution Criteria	CaBLAM outliers	40	3.86%	Goal: <1.0%
	CA Geometry outliers	28	2.71%	Goal: <0.5%

Summary of MolProbity results for the 3D model of full-length GluE7_Bbe. The model was generated by RaptorX using PDB entries 4u4g (chain A) and 3o4x:E as automatically selected templates for residues Met1–Val948 and Asp949–Val1038, respectively (*P*-values: 4.69e-17 and

9.39e-02, respectively), and further refined with ModRefiner. The tables presented above indicate model quality before and after refinement, respectively. Note the improvement of the model upon refinement, as indicated by the clashscore, Ramachandran, and overall MolProbity values.

*Clashscore is the number of serious steric overlaps (> 0.4 Å) per 1000 atoms. 100th percentile is the best among structures of comparable resolution; 0th percentile is the worst. For clashscore the comparative set of structures was selected in 2004, for MolProbity score in 2006.

^MolProbity score combines the clashscore, rotamer, and Ramachandran evaluations into a single score, normalized to be on the same scale as X-ray resolution.

All-Atom Contacts	Clashscore, all atoms:	7.17		86th percentile* (N=1784, all resolutions)
Protein Geometry	Poor rotamers	1	0.44%	Goal: <0.3%
	Favored rotamers	224	97.82%	Goal: >98%
	Ramachandran outliers	3	1.16%	Goal: <0.05%
	Ramachandran favored	246	94.98%	Goal: >98%
	MolProbity score#	1.74		88th percentile* (N=27675, 0Å - 99Å)
	Cβ deviations >0.25Å	4	1.65%	Goal: 0
	Bad bonds:	2 / 2168	0.09%	Goal: 0%
	Bad angles:	37 / 2918	1.27%	Goal: <0.1%
Peptide Omegas	Cis Prolines:	2 / 8	25.00%	Expected: ≤1 per chain, or ≤5%
	Cis nonProlines:	1 / 252	0.40%	Goal: <0.05%

Summary of MolProbity results for the 3D model of glycine-bound ligand-binding domain of GluE1_Bba. The model was generated by SWISS-MODEL based on the best available template, the atomic-resolution structure of rat GluA2 with bound glutamate (PDB 4yu0; refined at 1.26 Å resolution). At the light of the model quality, no further refinement was performed.

*Clashscore is the number of serious steric overlaps (> 0.4 Å) per 1000 atoms.100th percentile is the best among structures of comparable resolution; 0th percentile is the worst. For clashscore the comparative set of structures was selected in 2004, for MolProbity score in 2006.

#MolProbity score combines the clashscore, rotamer, and Ramachandran evaluations into a single score, normalized to be on the same scale as X-ray resolution.
