# Supplementary material for: Metazoan evolution of glutamate receptors reveals unreported phylogenetic groups and divergent lineage-specific events
Source: eLife. 2018 Nov 22;7:e35774. doi: 10.7554/eLife.35774 (PMC6307864; doi:10.7554/eLife.35774)
Supplement: Transparent reporting form [file elife-35774-transrepform.pdf]

eLIFE
1st Floor
24 Hills Road
Cambridge CB2 1JP, UK
P 01223 855340
W elifesciences.org
T @elife

eLife's transparent reporting form

We encourage authors to provide detailed information *within their submission* to facilitate the interpretation and replication of experiments. Authors can upload supporting documentation to indicate the use of appropriate reporting guidelines for health-related research (see EQUATOR Network), life science research (see the BioSharing Information Resource), or the ARRIVE guidelines for reporting work involving animal research. Where applicable, authors should refer to any relevant reporting standards documents in this form.

If you have any questions, please consult our Journal Policies and/or contact us: editorial@elifesciences.org.

Sample-size estimation

- You should state whether an appropriate sample size was computed when the study was being designed
- You should state the statistical method of sample size computation and any required assumptions
- If no explicit power analysis was used, you should describe how you decided what sample (replicate) size (number) to use

Please outline where this information can be found within the submission (e.g., sections or figure legends), or explain why this information doesn't apply to your submission:

Quantitative Gene Expression Analysis (qPCR):
Sample size (number of animals) was decided empirically based on the total RNA yield and RNA quality obtained after performing preliminary experiments with amphioxus samples (see Replicates section).

Information on sample size is provided in the Materials and Methods section:

'RNA isolation, cDNA synthesis and quantitative gene expression (qPCR).'

Replicates

- You should report how often each experiment was performed
- You should include a definition of biological versus technical replication
- The data obtained should be provided and sufficient information should be provided to indicate the number of independent biological and/or technical replicates
- If you encountered any outliers, you should describe how these were handled
- Criteria for exclusion/inclusion of data should be clearly stated
- High-throughput sequence data should be uploaded before submission, with a private link for reviewers provided (these are available from both GEO and ArrayExpress)

Please outline where this information can be found within the submission (e.g., sections or figure legends), or explain why this information doesn't apply to your submission:

eLife Sciences Publications, Ltd is a limited liability non-profit non-stock corporation incorporated in the State of Delaware, USA, with company number 5030732, and is registered in the UK with company number FC030576 and branch number BR015634 at the address 1st Floor, 24 Hills Road, Cambridge CB2 1JP | August 2014

Quantitative Gene Expression Analysis (qPCR):
The information on number of experiments performed and biological vs. technical replicates done can be found in the Materials and Methods section:

'RNA isolation, cDNA synthesis and quantitative gene expression (qPCR).'

qPCR analysis was done with total RNA isolated from either the entire animal or from the nerve chord. Preliminary experiments to decide what method rendered the best RNA yield and quality were performed in whole animals using TriReagent (Sigma), Maxwell 16 Total RNA Purification Kit (Promega) and RNAeasy mini kit (Qiagen), following manufacturer's instructions. TriReagent extraction method was chosen as the best method according to the nanodrop and Bioanalyzer data and both whole animal and nerve cord samples were further processed using the same method.

No outliers were encountered. This is stated in the above mentioned Materials and methods section.
None of the qPCR data was excluded. This is stated in the above mentioned Materials and methods section.

We do not present high-throughput data.

Statistical reporting

- Statistical analysis methods should be described and justified
- Raw data should be presented in figures whenever informative to do so (typically when N per group is less than 10)
- For each experiment, you should identify the statistical tests used, exact values of N, definitions of center, methods of multiple test correction, and dispersion and precision measures (e.g., mean, median, SD, SEM, confidence intervals; and, for the major substantive results, a measure of effect size (e.g., Pearson's r, Cohen's d)
- Report exact p-values wherever possible alongside the summary statistics and 95% confidence intervals. These should be reported for all key questions and not only when the p-value is less than 0.05.

Please outline where this information can be found within the submission (e.g., sections or figure legends), or explain why this information doesn't apply to your submission:

Phylogenetics:

This information can be found in the Materials and methods section: 'Phylogenetic analyses'; as well as in the figure legends of **Figure 1**, **Figure 1 – figure supplement 1, Figure 1 – figure supplement 2, Figure 1 – figure supplement 3, Figure 4 and Figure 4 – figure supplement 1**.

Quantitative Gene Expression Analysis (qPCR):

This information can be found in Materials and methods section: 'RNA isolation, cDNA synthesis and quantitative gene expression (qPCR)' and in the legends from **Figure 2** and **Figure 4 – figure supplement 2**.

(For large datasets, or papers with a very large number of statistical tests, you may upload a single table file with tests, Ns, etc., with reference to sections in the manuscript.)

Group allocation

- Indicate how samples were allocated into experimental groups (in the case of clinical studies, please specify allocation to treatment method); if randomization was used, please also state if restricted randomization was applied
- Indicate if masking was used during group allocation, data collection and/or data analysis

Please outline where this information can be found within the submission (e.g., sections or figure legends), or explain why this information doesn't apply to your submission:

In this manuscript we do not make use of experimental groups, such as 'case vs. control' or 'vehicle vs. treated' groups. We do statistically compare gene expression levels between the entire animal (amphioxus) and its nerve chord in order to elucidate if glutamate receptor gene expression occurs primarily in the nervous tissue. This is all done with wild-type specimens. For this particular experiment we consider that there is no need to organize the samples into experimental groups.

Additional data files ("source data")

- We encourage you to upload relevant additional data files, such as numerical data that are represented as a graph in a figure, or as a summary table

eLife Sciences Publications, Ltd is a limited liability non-profit non-stock corporation incorporated in the State of Delaware, USA, with company number 5030732, and is registered in the UK with company number FC030576 and branch number BR015634 at the address 1st Floor, 24 Hills Road, Cambridge CB2 1JP | August 2014

- Where provided, these should be in the most useful format, and they can be uploaded as "Source data" files linked to a main figure or table
- Include model definition files including the full list of parameters used
- Include code used for data analysis (e.g., R, MatLab)
- Avoid stating that data files are "available upon request"

Please indicate the figures or tables for which source data files have been provided:

We have included the following five source data files:

Figure 1 – source data 4: Sequence alignment used to reconstruct phylogenetic evolution of iGluRs. Related to Figure 1 and Figure 1 – figure supplement 1.

Figure 1 – source data 5: Sequence alignment used to reconstruct phylogenetic evolution of AMPA and Kainate classes in protostomes. Related to Figure 1 – figure supplement 3 and Figure 1 – figure supplement 4.

Figure 2 – source data 1: qPCR values obtained for the expression analysis of amphioxus iGluRs. Related to Figure 2a.

Figure 4 – source data 3: Sequence alignment used to reconstruct phylogenetic evolution of mGluRs. Related to Figure 4 and Figure 4 – figure supplement 1.

Figure 4 – source data 4: qPCR values obtained for the expression analysis of amphioxus iGluRs. Related to Figure 4 – figure supplement 3b.
